# Association between subclinical atherosclerosis and cardiac structure and function—results from the UK Biobank Study

**DOI:** 10.1093/ehjimp/qyad010

**Published:** 2023-09-19

**Authors:** Judit Simon, Kenneth Fung, Zahra Raisi-Estabragh, Nay Aung, Mohammed Y Khanji, Emese Zsarnóczay, Béla Merkely, Patricia B Munroe, Nicholas C Harvey, Stefan K Piechnik, Stefan Neubauer, Paul Leeson, Steffen E Petersen, Pál Maurovich-Horvat

**Affiliations:** MTA-SE Cardiovascular Imaging Research Group, Medical Imaging Centre, Semmelweis University, Üllői út 78, H-1082 Budapest, Hungary; Heart and Vascular Center, Semmelweis University, Budapest, Hungary, Városmajor u 68, H-1122 Budapest, Hungary; William Harvey Research Institute, NIHR Barts Biomedical Research Centre, Queen Mary University of London, London EC1M 6BQ, United Kingdom; Barts Heart Centre, St Bartholomew’s Hospital, Barts Health NHS Trust, West Smithfield, London EC1A 7BE, United Kingdom; William Harvey Research Institute, NIHR Barts Biomedical Research Centre, Queen Mary University of London, London EC1M 6BQ, United Kingdom; Barts Heart Centre, St Bartholomew’s Hospital, Barts Health NHS Trust, West Smithfield, London EC1A 7BE, United Kingdom; William Harvey Research Institute, NIHR Barts Biomedical Research Centre, Queen Mary University of London, London EC1M 6BQ, United Kingdom; Barts Heart Centre, St Bartholomew’s Hospital, Barts Health NHS Trust, West Smithfield, London EC1A 7BE, United Kingdom; William Harvey Research Institute, NIHR Barts Biomedical Research Centre, Queen Mary University of London, London EC1M 6BQ, United Kingdom; Barts Heart Centre, St Bartholomew’s Hospital, Barts Health NHS Trust, West Smithfield, London EC1A 7BE, United Kingdom; Barts Health NHS Trust, Newham University Hospital, Glen Road, Plaistow, London E1 1BB, United Kingdom; MTA-SE Cardiovascular Imaging Research Group, Medical Imaging Centre, Semmelweis University, Üllői út 78, H-1082 Budapest, Hungary; Heart and Vascular Center, Semmelweis University, Budapest, Hungary, Városmajor u 68, H-1122 Budapest, Hungary; Heart and Vascular Center, Semmelweis University, Budapest, Hungary, Városmajor u 68, H-1122 Budapest, Hungary; William Harvey Research Institute, NIHR Barts Biomedical Research Centre, Queen Mary University of London, London EC1M 6BQ, United Kingdom; MRC Lifecourse Epidemiology Unit, University of Southampton, Southampton SO16 6YD, United Kingdom; National Institute for Health Research, Oxford Biomedical Research Centre, Division of Cardiovascular Medicine, Radcliffe Department of Medicine, University of Oxford, Oxford OX3 9DU, United Kingdom; National Institute for Health Research, Oxford Biomedical Research Centre, Division of Cardiovascular Medicine, Radcliffe Department of Medicine, University of Oxford, Oxford OX3 9DU, United Kingdom; Oxford Cardiovascular Clinical Research Facility, Division of Cardiovascular Medicine, Radcliffe Department of Medicine, University of Oxford, Level 1, John Radcliffe Hospital, Oxford OX3 9DU, United Kingdom; William Harvey Research Institute, NIHR Barts Biomedical Research Centre, Queen Mary University of London, London EC1M 6BQ, United Kingdom; Barts Heart Centre, St Bartholomew’s Hospital, Barts Health NHS Trust, West Smithfield, London EC1A 7BE, United Kingdom; MTA-SE Cardiovascular Imaging Research Group, Medical Imaging Centre, Semmelweis University, Üllői út 78, H-1082 Budapest, Hungary; Heart and Vascular Center, Semmelweis University, Budapest, Hungary, Városmajor u 68, H-1122 Budapest, Hungary

**Keywords:** cardiac structure and function, carotid, heart failure, MRI

## Abstract

**Aims:**

Heart failure (HF) is a major health problem and early diagnosis is important. Atherosclerosis is the main cause of HF and carotid intima-media thickness (IMT) is a recognized early measure of atherosclerosis. This study aimed to investigate whether increased carotid IMT is associated with changes in cardiac structure and function in middle-aged participants of the UK Biobank Study without overt cardiovascular disease.

**Methods and results:**

Participants of the UK Biobank who underwent CMR and carotid ultrasound examinations were included in this study. Patients with heart failure, angina, atrial fibrillation, and history of myocardial infarction or stroke were excluded. We used multivariable linear regression models adjusted for age, sex, physical activity, body mass index, body surface area, hypertension, diabetes, smoking, ethnicity, socioeconomic status, alcohol intake, and laboratory parameters. In total, 4301 individuals (61.6 ± 7.5 years, 45.9% male) were included. Multivariable linear regression analyses showed that increasing quartiles of IMT was associated with increased left and right ventricular (LV and RV) and left atrial volumes and greater LV mass. Moreover, increased IMT was related to lower LV end-systolic circumferential strain, torsion, and both left and right atrial ejection fractions (all *P* < 0.05).

**Conclusion:**

Increased IMT showed an independent association over traditional risk factors with enlargement of all four cardiac chambers, decreased function in both atria, greater LV mass, and subclinical LV dysfunction. There may be additional risk stratification that can be derived from the IMT to identify those most likely to have early cardiac structural/functional changes.

## Graphical Abstract

**Graphical Abstract:**
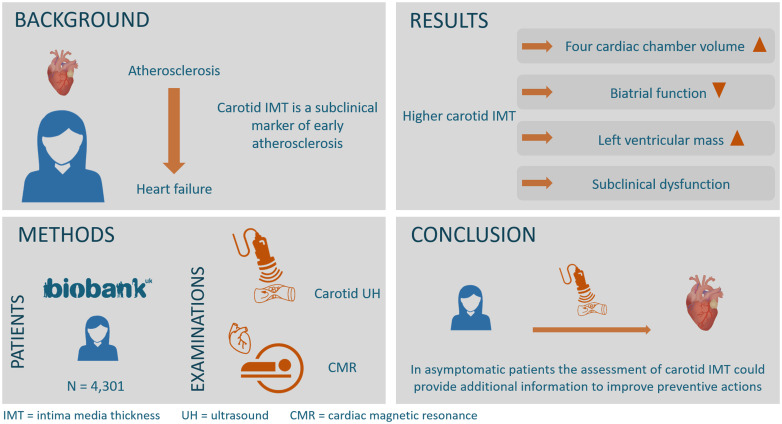

## Introduction

Since heart failure (HF) is associated with high morbidity, mortality, and health-care costs, timely diagnosis of subclinical cardiac dysfunction is an important aspect of cardiovascular (CV) preventive strategies. Myocardial strain has been shown to be a reliable marker of systolic dysfunction, especially in the early stages, when ejection fraction (EF) is not able to depict subtle alterations in cardiac function.^1^ However, strain measurements with speckle tracking echocardiography can be challenging due to issues regarding standardization across the different vendors and low agreement with cardiac magnetic resonance (CMR)-based strain analysis. Therefore other CV biomarkers might be important for the early detection of HF.

Atherosclerosis and its sequelae are one of the main causes of HF in the developing world.^2^ Carotid intima-media thickness (IMT) is a well-established subclinical marker of early atherosclerosis.^3^ Even if its use is not recommended for risk stratification in clinical guidelines, previous studies have shown that IMT is associated with future CV events such as myocardial infarction, angina, coronary intervention, stroke, or transient ischaemic attack.^4–7^ Due to its simplicity and excellent correlation with histology, it is widely used in everyday clinical practice.^8^ In a recent study of 1161 participants, increased carotid IMT was associated with impaired left ventricular (LV) and left atrial (LA) strain parameters, as assessed by 2-dimensional speckle-tracking echocardiography.^9^ Due to its superior spatial resolution, CMR is considered as the gold standard for the evaluation of LV structural and functional parameters.^10^ Moreover, it allows the precise assessment of the right ventricle (RV) and the atria.^11^

We aimed to study the association between carotid IMT and cardiac structural and functional parameters as assessed by CMR. Moreover, we aimed to investigate the association of IMT with subclinical LV dysfunction in a large UK cohort of participants without clinically manifested CV disease.

## Methods

### Study sample

The UK Biobank is a prospective cohort study that collected questionnaire data, physical measurements, and biological samples from half a million 40–69 year-old individuals in the United Kingdom.^12^ In total, 100 000 participants are being recalled to undergo comprehensive imaging of the brain, heart, whole body, carotid artery, bone, and joints. Imaging of the heart is performed by CMR and carotid IMT was assessed by carotid ultrasound.^13,14^ CMR examinations of 5065 consecutive participants have already been manually contoured and analysed. These participants formed the recruitment cohort for our analysis. Exclusion criteria of the current study were poor image quality, HF, angina, prior myocardial infarction, and stroke based on the criteria described previously.^13,14^

This study was covered by the ethical approval for UK Biobank studies from the National Health Service (NHS) National Research Ethics Service on 17th June 2011 (Ref 11/NW/0382), extended 10th May 2016 (Ref 16/NW/0274), and extended on 18 June 2021 (Ref 21/NW/0157) with written informed consent by all UK Biobank participants.

### Measurement of baseline covariates and potential cofounders

Covariates were determined from patient interviews or touchscreen questionnaires. These included age, sex, ethnicity, Townsend deprivation index (a socio-economic measure based on area of residence), weight and height, and comorbidities. Moreover, lifestyle factors such as physical activity (expressed as metabolic equivalent of task (MET) score), alcohol intake frequency (never, special occasions only, 1–3 times per month, 1–2 times per week, 3–4 times per week, and daily or almost daily), and smoking status (never smoker and previous or current smoker) were also ascertained. The following serum biochemistry measures from blood collected at the baseline visit were considered as potential mediators: total cholesterol, lipoprotein A, apolipoprotein A and B, triglyceride, glycated haemoglobin, urate, and C-reactive protein. Detailed questions of the UK Biobank questionnaires can be found in UK Biobank Data Showcase (https://www.ukbiobank.ac.uk/data-showcase/).

### Carotid IMT measurements

Carotid IMT was measured by 2-dimensional ultrasound (CardioHealth Station, Panasonic Healthcare Corporation of North America, Newark, NJ, USA) at two pre-defined angles for each carotid giving a total of four carotid IMT values: right 150°, right 120°, left 210°, and left 240°. A mean, maximum, and minimum of the carotid IMT tracking was recorded for each carotid for each angle of acquisition. The average of the four mean measures was calculated and incorporated into our analysis. The detailed protocol has previously been published.^14^

### CMR protocol and image analysis

The UK Biobank CMR protocol has been described in detail previously.^15,16^ Briefly, all examinations was performed on a 1.5 Tesla scanner (MAGNETOM Aera, Syngo Platform VD13A, Siemens Healthcare, Erlangen, Germany). For cardiac function, long-axis cines and a complete short-axis stack of balanced steady-state free precession (bSSFP) cines were acquired covering the LV and RV.

Analysis of the LV, RV, LA, and right atrium (RA) for all CMR examinations were performed across two core laboratories in London and Oxford according to pre-approved standard operating procedures using dedicated post-processing software (cvi42, Version 5.1.1, Circle Cardiovascular Imaging Inc., Calgary, Canada). LV papillary muscles were included in the LV end-diastolic volume (LVEDV) and end-systolic volume (LVESV). For the measurement of RV parameters, manual tracing of the end-diastolic and end-systolic endocardial borders was carried out in the short-axis view. Thin-walled structures with no trabeculation were excluded and volumes below the pulmonary valves were included as part of the RV. LA and RA end-diastolic volumes (EDV) and end-systolic volumes (ESV), stroke volumes (SV), and EF were calculated based on the manually traced endocardial atrial contours in a four-chamber view. Detailed descriptions of analysis methodology, including exemplar contours and intra- and inter-observer variability, have been previously described.^16^

Semi-automated analysis of tagged cine images was performed with CIM software (CAROTIS IMTag2D v8.1.5 software, Auckland MRI Research Group, New Zealand).^17^ A grid was aligned automatically to the myocardial tagging planes at end-diastole. End-systole was determined visually, and tags were manually adjusted at key phases during the cardiac cycle including the end-systolic and last frame. Global circumferential strain (GCS) was calculated from the motion of the intersected tag lines at basal, mid, and apical levels. Torsion, the wringing motion induced by the contracting myofibers in the LV wall during systole has been shown to be a sensitive marker of myocardial dysfunction.^18^ Torsion was calculated from the basal and apical strain measures, as previously described.^19^ In those cases when a basal or apical slice was missing or not analyzable, torsion was calculated between the mid-ventricular and the other available slice.

### Data analysis and statistics

Summary statistics for independent variables were calculated as means and standard deviation (SD) for continuous variables. Categorical variables were expressed as frequencies and percentages. Carotid IMT was handled as a categorical variable using quartiles. To assess the relationship between carotid IMT and cardiac anatomy and function, the various CMR parameters were analysed using unadjusted and multivariable linear regression analyses. In order to test the mediating effects of the potential confounders and mediators, we built two sets of models: Model 1: Adjustment was made for age, sex, physical activity, body mass index (BMI), body surface area (BSA), hypertension, diabetes, smoking, non-European ethnicity, Townsend deprivation index, alcohol intake frequency, total cholesterol; Model 2: Adjustment was made for Model 1 + lipoprotein A, apolipoprotein A and B, triglyceride, glycated haemoglobin, urate, and C-reactive protein. Definitions for these covariates were previously described.^20,21^ Statistical analysis was performed using R (version 4.0.3) Statistical Software.^22^

## Results

### Study population

A number of exclusions is listed in Supplementary data online, *Figure S1*. After exclusion, 4301 participants were included in our analysis. Mean age was 61.6 ± 7.5 years and 1976 (45.9%) were male. The average of the mean carotid IMT was 673.6 ± 123.7 micrometer. Participants were grouped into four carotid IMT categories using quartiles: quartile 1 (<584 micrometer), quartile 2 (584–652 micrometer), quartile 3 (653–746 micrometer), and quartile 4 (>746 micrometer). Almost all CV risk factors were positively associated with gradually increasing carotid IMT. Clinical characteristics of the study population by IMT quartiles are reported in *Table 1*.

**Table 1:** Clinical characteristics

	Quartiles of carotid IMT				
	Carotid IMT quartile 1 (<584 micrometer; *n* = 1082)	Carotid IMT quartile 2 (584–652 micrometer; *n* = 1072)	Carotid IMT quartile 3 (653–746 micrometer; *n* = 1076)	Carotid IMT quartile 4 (>746 micrometer; 1071)	*P*
Age (years)	56.9 ± 6.9	60.5 ± 7.1	63.2 ± 6.7	65.8 ± 6.2	<0.001
Male, *n* (%)	389 (35.9)	439 (41.0)	480 (44.6)	668 (62.4)	<0.001
Non-European ethnicity, *n* (%)	47 (4.4)	29 (2.7)	26 (2.4)	23 (2.1)	0.011
Townsend deprivation index	−1.83 ± 2.84	−1.91 ± 2.72	−2.02 ± 2.58	−2.18 ± 2.60	0.018
BMI (kg/m^2^)	25.9 ± 4.2	26.4 ± 4.2	26.5 ± 4.0	26.9 ± 4.2	<0.001
BSA (m^2^)	1.81 ± 0.2	1.84 ± 0.2	1.84 ± 0.2	1.89 ± 0.2	<0.001
Hypertension, *n* (%)	177 (16.4)	242 (22.6)	289 (26.9)	366 (34.2)	<0.001
Diabetes mellitus, *n* (%)	29 (2.7)	45 (4.3)	43 (4.1)	59 (5.6)	0.012
Total cholesterol level (mmol/L)	5.64 ± 1.05	5.76 ± 1.08	5.78 ± 1.05	5.80 ± 1.06	0.004
Physical activity (METs)	2460.0 ± 2443.9	2619.5 ± 2407.3	2747.9 ± 2618.2	2662.7 ± 2381.1	0.062
Alcohol intake frequency					
Never, *n* (%)	70 (6.5)	63 (6.0)	73 (6.9)	52 (4.9)	<0.001
Special occasions only, *n* (%)	138 (12.9)	113 (10.7)	97 (9.1)	117 (11.0)	
1–3 times/month, *n* (%)	134 (12.5)	129 (12.2)	101 (9.5)	116 (10.9)	
1–2 times/week, *n* (%)	314 (29.3)	271 (25.6)	294 (27.7)	256 (24.1)	
3–4 times/week, *n* (%)	258 (24.1)	288 (27.2)	282 (26.6)	289 (27.2)	
Daily or almost daily, *n* (%)	157 (14.7)	194 (18.3)	215 (20.2)	233 (21.9)	
Previous or current smoker, *n* (%)	340 (31.8)	400 (37.9)	408 (38.5)	480 (45.2)	<0.001

Continuous values are expressed as mean ± standard deviation and categorical variables are expressed as frequencies and percentages.

BMI, body mass index; BSA, body surface area; IMT, intima-media thickness; MET, metabolic equivalent of task.

### Association between carotid IMT and LV structure and function

**Figure 1:**
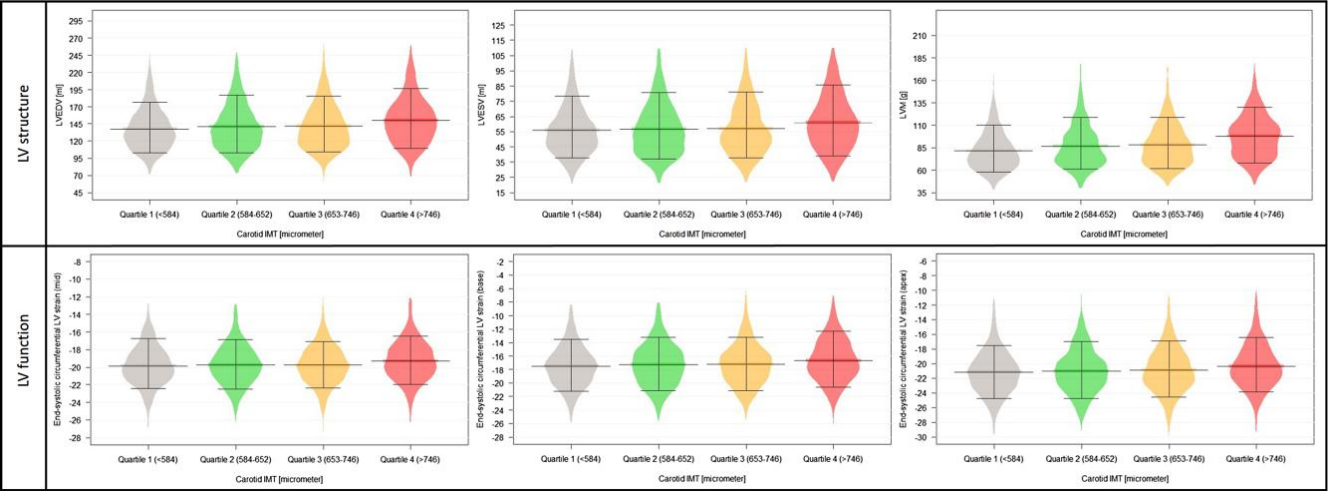
Distribution of LV structural and functional parameters among carotid IMT quartiles. *P < 0.05. Abbreviations: IMT, intima-media thickness; LVEDV, left ventricular end-diastolic volume; LVESV, left ventricular end-systolic volume; LVM, left ventricular mass.

Significantly increasing LVEDV, LVESV, left ventricular stroke volume (LVSV), and left ventricular mass (LVM) values were measured by quartiles reflecting increasing carotid IMT (all *P* < 0.001). While left ventricular ejection fraction (LVEF) values did not differ among the carotid IMT quartiles, significant worsening in the end-systolic circumferential strain at the basal, mid, and apical levels of the heart were observed together with significantly increasing torsion (all *P* < 0.001). The distribution of the above-mentioned LV parameters by IMT quartiles can be seen in *Figure 1*. In fully adjusted linear regression models, compared to the reference quartile 1 of IMT, all higher carotid IMT quartiles were associated with bigger LVEDV and LVM values, and IMT quartile 4 was independently associated with higher LVESV, LVSV, end-systolic circumferential LV strain, and torsion values. There was no association with LVEF in any higher carotid IMT quartiles. Results of the uni- and multivariable analyses can be seen in *Table 2*.

**Table 2:** Association between carotid IMT and LV structural and functional parameters

	Mean value ± SD	Unadjusted		Model 1		Model 2	
		ß (95%CI)	*P*	ß (95%CI)	*P*	ß (95%CI)	*P*
LVEDV (ml)							
Carotid IMT quartile 1	137.9 ± 31.2	Reference		Reference		Reference	
Carotid IMT quartile 2	142.2 ± 36.4	4.32 (1.41–7.23)	0.004	4.41 (2.01–6.81)	<0.001	3.70 (0.77–6.63)	0.013
Carotid IMT quartile 3	142.2 ± 33.5	4.35 (1.44–7.26)	0.003	4.91 (2.39–7.42)	<0.001	3.81 (0.69–6.93)	0.017
Carotid IMT quartile 4	150.9 ± 34.4	13.07 (10.15–15.98)	<0.001	9.45 (6.78–12.13)	<0.001	9.53 (6.26–12.80)	<0.001
LVESV (ml)							
Carotid IMT quartile 1	56.5 ± 18.8	Reference		Reference		Reference	
Carotid IMT quartile 2	58.1 ± 21.8	1.61 (−0.11–3.33)	0.066	2.03 (0.49–3.58)	0.010	1.86 (−0.08–3.81)	0.060
Carotid IMT quartile 3	58.2 ± 19.6	1.75 (0.03–3.48)	0.046	2.36 (0.74–3.99)	0.004	2.06 (−0.01–4.13)	0.052
Carotid IMT quartile 4	62.4 ± 19.9	5.91 (4.19–7.63)	<0.001	4.08 (2.35–5.80)	<0.001	4.48 (2.31–6.65)	<0.001
LVM (g)							
Carotid IMT quartile 1	81.4 ± 21.3	Reference		Reference		Reference	
Carotid IMT quartile 2	87.0 ± 24.8	5.59 (3.56–7.63)	<0.001	4.43 (2.95–5.90)	<0.001	3.37 (1.55–5.18)	<0.001
Carotid IMT quartile 3	88.5 ± 23.8	7.08 (5.04–9.12)	<0.001	5.16 (3.61–6.71)	<0.001	4.46 (2.53–6.40)	<0.001
Carotid IMT quartile 4	98.4 ± 24.9	16.97 (14.93–19.01)	<0.001	10.05 (8.41–11.70)	<0.001	9.71 (7.68–11.73)	<0.001
LVSV (ml)							
Carotid IMT quartile 1	81.4 ± 17.4	Reference		Reference		Reference	
Carotid IMT quartile 2	84.1 ± 20.1	2.71 (1.07–4.35)	0.001	2.37 (0.90–3.84)	0.002	1.83 (0.05–3.60)	0.043
Carotid IMT quartile 3	84.0 ± 18.9	2.59 (0.95–4.23)	0.002	2.55 (1.01–4.09)	0.001	1.75 (−0.14–3.64)	0.070
Carotid IMT quartile 4	88.5 ± 20.0	7.17 (5.52–8.81)	<0.001	5.40 (9.76–7.04)	<0.001	5.09 (3.11–7.07)	<0.001
LVEF (%)							
Carotid IMT quartile 1	59.4 ± 6.0	Reference		Reference		Reference	
Carotid IMT quartile 2	59.7 ± 6.4	0.22 (−0.32–0.76)	0.422	0.01 (−0.58–0.60)	0.976	0.00 (−0.72–0.72)	0.998
Carotid IMT quartile 3	59.5 ± 6.4	0.11 (−0.43–0.65)	0.695	−0.12 (−0.74–0.50)	0.705	−0.31 (−1.07–0.46)	0.430
Carotid IMT quartile 4	59.1 ± 6.6	−0.33 (−0.87–0.21)	0.235	−0.06 (−0.71–0.60)	0.864	−0.35 (−1.15–0.45)	0.392
End-systolic circumferential LV strain (base)							
Carotid IMT quartile 1	−17.5 ± 3.1	Reference		Reference		Reference	
Carotid IMT quartile 2	−17.2 ± 3.2	0.27 (−0.05–0.59)	0.102	0.20 (−1.53–0.55)	0.269	0.32 (−4.20–0.81)	0.077
Carotid IMT quartile 3	−17.2 ± 3.1	0.29 (−0.03–0.61)	0.076	0.09 (−2.78–0.46)	0.631	0.40 (−5.44–0.85)	0.085
Carotid IMT quartile 4	−16.6 ± 3.3	0.90 (0.58–1.22)	<0.001	0.47 (0.07–0.86)	0.020	0.74 (0.26–1.21)	0.002
End-systolic circumferential LV strain (mid)							
Carotid IMT quartile 1	−19.8 ± 2.3	Reference		Reference		Reference	
Carotid IMT quartile 2	−19.7 ± 2.4	0.15 (−0.07–0.37)	0.183	0.08 (−0.15–0.31)	0.519	0.17 (−0.11–0.44)	0.233
Carotid IMT quartile 3	−19.7 ± 2.3	0.10 (−0.12–0.32)	0.375	−0.05 (−0.29–0.19)	0.670	0.11 (−1.83–0.40)	0.470
Carotid IMT quartile 4	−19.2 ± 2.4	0.59 (0.38–0.81)	<0.001	0.15 (−0.11–0.41)	0.261	0.32 (0.01–0.62	0.043
End-systolic circumferential LV strain (apex)							
Carotid IMT quartile 1	−21.2 ± 3.0	Reference		Reference		Reference	
Carotid IMT quartile 2	−20.9 ± 3.1	0.23 (−0.07–0.53)	0.133	0.18 (−0.13–0.50)	0.254	0.26 (−0.12–0.63)	0.185
Carotid IMT quartile 3	−20.8 ± 3.1	0.30 (0.00–0.60)	0.050	0.11 (−0.22–0.44)	0.505	0.39 (−0.01–0.79)	0.056
Carotid IMT quartile 4	−20.3 ± 3.1	0.85 (0.54–1.15)	<0.001	0.35 (0.00–0.71)	0.052	0.45 (0.02–0.87)	0.041
End-systolic torsion (°)							
Carotid IMT quartile 1	7.3 ± 1.9	Reference		Reference		Reference	
Carotid IMT quartile 2	7.7 ± 2.1	0.37 (0.18–0.56)	<0.001	0.24 (0.04–0.45)	0.019	0.23 (−0.01–0.47)	0.061
Carotid IMT quartile 3	7.7 ± 2.0	0.42 (0.23–0.61)	<0.001	0.16 (−0.06–0.37)	0.148	0.14 (−0.12–0.39)	0.299
Carotid IMT quartile 4	7.8 ± 2.1	0.56 (0.37–0.76)	<0.001	0.30 (0.07–0.53)	0.010	0.33 (0.06–0.60)	0.018

Model 1: Adjusted for age, sex, physical activity, BMI, BSA, hypertension, diabetes, smoking, ethnicity, Townsend deprivation index, alcohol intake frequency, and total cholesterol.

Model 2: Adjusted for Model 1 + lipoprotein A, apolipoprotein A and B, triglyceride, glycated haemoglobin, urate, and C-reactive protein.

CI, confidence interval; IMT, intima-media thickness; LVEDV, left ventricular end-diastolic volume; LVEF, left ventricular ejection fraction; LVESV, left ventricular end-systolic volume; LVM, left ventricular mass; LVSV, left ventricular stroke volume.

### Association of carotid IMT with RV and atrial structure and function

**Figure 2:**
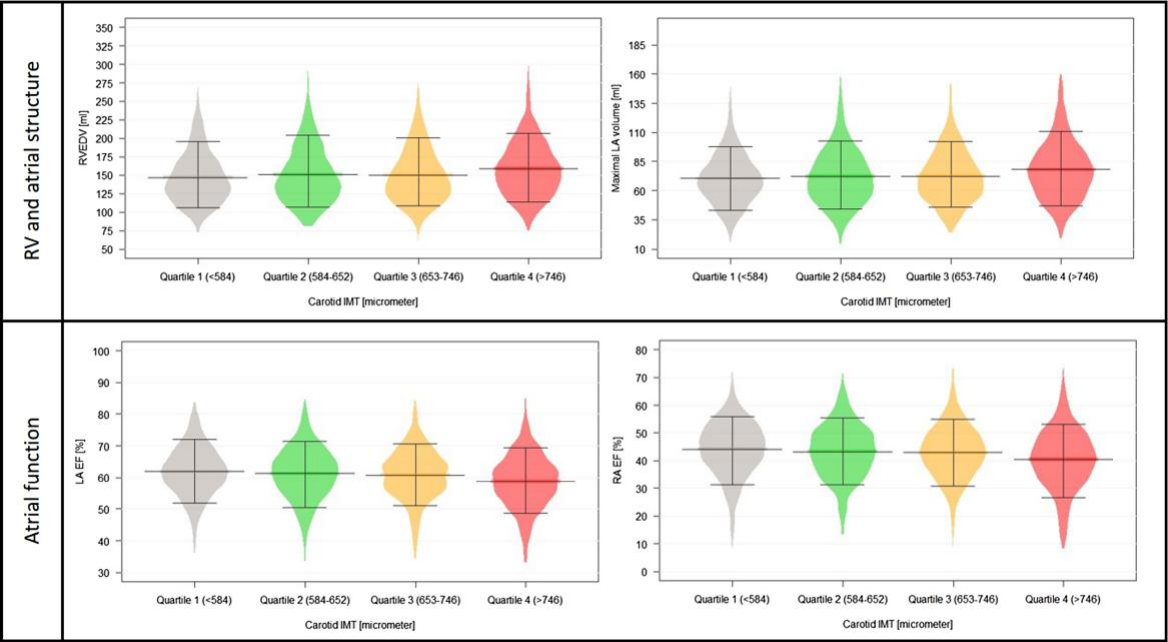
Distribution of RV and atrial structural and functional parameters among carotid IMT quartiles. *P < 0.05. Abbreviations: EF, ejection fraction; IMT, intima-media thickness; LA, left atrium; RA, right atrium; RVEDV, right ventricular end-diastolic volume.

Those having the highest IMT values had significantly higher right ventricular end-diastolic volume (RVEDV) (158.9 ± 37.7 vs. 146.8 ± 34.6 mL, *P* < 0.001), right ventricular end-systolic volume (RVESV) (70.2 ± 22.9 vs. 65.2 ± 21.3 mL, *P* < 0.001), maximal LA (79.7 ± 29.4 vs. 70.7 ± 21.8 mL, *P* < 0.001), and RA (82.9 ± 29.0 vs. 76.0 ± 22.8 mL, *P* < 0.001) volumes, as compared to quartile 1. Regarding functional parameters, while there were no differences among the groups in right ventricular ejection fraction (RVEF), gradually decreasing LA EF and RA EF values were measured parallel with higher carotid IMT (both *P* < 0.001). The distribution of significantly increasing RV and atrial structural and functional parameters can be seen in *Figure 2*. After adjustment for all CV risk factors and pertinent laboratory parameters, association with RVEDV, maximal LA volume, and reduced atrial function remained significant. Results of the uni- and multivariable analyses can be seen in *Table 3*.

**Table 3:** Association of carotid IMT with RV and atrial structure and function

	Mean value ± SD	Unadjusted		Model 1		Model 2	
		ß (95%CI)	*P*	ß (95%CI)	*P*	ß (95%CI)	*P*
RVEDV (ml)							
Carotid IMT quartile 1	146.8 ± 34.6	Reference		Reference		Reference	
Carotid IMT quartile 2	151.3 ± 39.8	4.51 (1.33–7.70)	0.005	3.89 (1.44–6.34)	0.002	3.00 (0.05–5.95)	0.046
Carotid IMT quartile 3	149.6 ± 36.0	2.78 (−0.41–5.97)	0.087	2.60 (0.03–5.17)	0.048	1.06 (−2.08–4.20)	0.509
Carotid IMT quartile 4	158.9 ± 37.7	12.14 (8.95–15.33)	<0.001	6.45 (3.72–9.18)	<0.001	6.08 (2.80–9.37)	<0.001
RVESV (ml)							
Carotid IMT quartile 1	65.2 ± 21.3	Reference		Reference		Reference	
Carotid IMT quartile 2	66.8 ± 23.5	1.59 (−0.32–3.51)	0.103	1.62 (0.04–3.21)	0.045	1.50 (−0.42–3.42)	0.126
Carotid IMT quartile 3	65.5 ± 21.7	0.31 (−1.61–2.23)	0.756	0.64 (−1.02–2.31)	0.449	0.11 (−1.94–2.15)	0.920
Carotid IMT quartile 4	70.2 ± 22.9	5.00 (3.07–6.92)	<0.001	1.97 (0.20–3.74)	0.029	2.13 (−0.01–4.28)	0.050
RVSV (ml)							
Carotid IMT quartile 1	81.6 ± 17.8	Reference		Reference		Reference	
Carotid IMT quartile 2	84.5 ± 20.2	2.91 (1.26–4.55)	<0.001	2.24 (0.81–3.67)	0.002	1.48 (−0.25–3.21)	0.095
Carotid IMT quartile 3	84.1 ± 18.9	2.49 (0.84–4.14)	0.003	1.95 (0.45–3.46)	0.011	0.96 (−0.88–2.81)	0.305
Carotid IMT quartile 4	88.8 ± 19.8	7.14 (5.50–8.79)	<0.001	4.47 (2.87–6.06)	<0.001	3.94 (2.01–5.87)	<0.001
RVEF (%)							
Carotid IMT quartile 1	56.2 ± 6.3	Reference		Reference		Reference	
Carotid IMT quartile 2	56.5 ± 6.3	0.32 (−0.24–0.87)	0.264	0.12 (−0.46–0.71)	0.675	−0.07 (−0.78–0.64)	0.839
Carotid IMT quartile 3	56.8 ± 6.6	0.61 (0.05–1.16)	0.033	0.36 (−0.26–0.97)	0.256	0.25 (−0.50–1.01)	0.511
Carotid IMT quartile 4	56.4 ± 6.6	0.21 (−0.34–0.77)	0.452	0.59 (−0.06–1.25)	0.073	0.32 (−0.47–1.12)	0.423
Maximal LA volume (ml)							
Carotid IMT quartile 1	70.7 ± 21.8	Reference		Reference		Reference	
Carotid IMT quartile 2	72.6 ± 24.6	1.93 (−0.23–4.10)	0.080	2.63 (0.38–4.88)	0.022	1.25 (−1.45–3.96)	0.364
Carotid IMT quartile 3	73.8 ± 23.3	2.04 (−0.14–4.21)	0.067	3.54 (1.16–5.91)	0.004	2.39 (−0.52–5.29)	0.107
Carotid IMT quartile 4	79.7 ± 29.4	9.00 (6.80–11.14)	<0.001	9.77 (7.25–12.29)	<0.001	8.85 (5.81–11.88)	<0.001
LA EF (%)							
Carotid IMT quartile 1	61.9 ± 8.2	Reference		Reference		Reference	
Carotid IMT quartile 2	60.8 ± 9.4	−1.15 (−2.00 to −0.33)	0.006	−0.73 (−1.61–0.15)	0.103	−0.99 (−2.09–0.11)	0.077
Carotid IMT quartile 3	60.4 ± 9.4	−1.50 (−2.30 to −0.66)	<0.001	−0.56 (−1.50–0.37)	0.235	−0.73 (−1.91–0.45)	0.226
Carotid IMT quartile 4	57.8 ± 10.6	−4.09 (−4.91 to −3.27)	<0.001	−2.18 (−3.17 to −1.20)	<0.001	−2.50 (−3.74 to −1.27)	<0.001
Maximal RA volume (ml)							
Carotid IMT quartile 1	76.0 ± 22.8	Reference		Reference		Reference	
Carotid IMT quartile 2	79.3 ± 26.7	3.27 (1.03–5.52)	0.004	1.70 (−0.43–3.82)	0.118	0.82 (1.73–3.37)	0.530
Carotid IMT quartile 3	78.2 ± 24.6	2.23 (−0.02–4.48)	0.063	0.00 (−2.24–2.24)	0.999	−0.65 (−3.39–2.08)	0.639
Carotid IMT quartile 4	82.9 ± 29.0	6.89 (4.64–9.14)	<0.001	0.62 (−1.76–3.00)	0.611	−0.20 (−3.07–2.66)	0.891
RA EF (%)							
Carotid IMT quartile 1	44.1 ± 10.1	Reference		Reference		Reference	
Carotid IMT quartile 2	43.1 ± 10.2	−0.94 (−1.85 to −0.02)	0.045	−0.23 (−1.19–0.73)	0.640	0.03 (1.13–1.20)	0.958
Carotid IMT quartile 3	42.7 ± 10.2	−1.34 (−2.26 to −0.42)	0.004	0.18 (−0.83–1.20)	0.723	0.31 (−0.94–1.56)	0.628
Carotid IMT quartile 4	40.1 ± 11.6	−4.00 (−4.92 to −3.09)	<0.001	−1.25 (−2.33 to −0.17)	0.023	−1.74 (−3.04 to −0.43)	0.009

Model 1: Adjusted for age, sex, physical activity, BMI, BSA, hypertension, diabetes, smoking, ethnicity, Townsend deprivation index, alcohol intake frequency, and total cholesterol.

Model 2: Adjusted for Model 1 + lipoprotein A, apolipoprotein A and B, triglyceride, glycated haemoglobin, urate, and C-reactive protein.

CI, confidence interval; EF, ejection fraction; IMT, intima-media thickness; LA, left atrium; RA, right atrium; RVEDV, right ventricular end-diastolic volume; RVEF, right ventricular ejection fraction; RVESV, right ventricular end-systolic volume; RVSV, right ventricular stroke volume.

## Discussion

To our knowledge, this is the largest study to investigate the association between carotid IMT measured by ultrasound and cardiac structural and functional parameters depicted by gold standard CMR in a largely middle-aged population without known overt CV disease. Our results suggest that higher carotid IMT is associated with subclinical dysfunction and changes in cardiac structure.

Previous studies reported an association between carotid IMT and incident HF. In a study of 4691 individuals without known myocardial infarction or stroke, high carotid IMT and C-reactive protein levels proved to be independent risk factors of future HF requiring hospitalization.^23^ In the Atherosclerosis Risk in Communities Study of 13 590 participants without baseline HF, carotid IMT was identified as an independent risk factor for HF development beyond risks explained by major CV risk factors and coronary heart disease.^24^ These results suggest that carotid IMT and HF might be associated with mechanisms other than myocardial ischaemia or infarction. Therefore, assessment of early alterations in the cardiac structure and function may be important in order to recognize the early stages of HF. However, literature data regarding the association between carotid IMT and subclinical LV dysfunction are limited. Echocardiography studies have found that higher carotid IMT is associated with impaired LV and LA strain independent of traditional CV risk factors, pertinent laboratory, and echocardiographic parameters.^9,25^

Myocardial deformation imaging can detect contractile dysfunction at an early stage in the majority of cardiac diseases. Circumferential myocardial strain represents the myocardial fibre shortening along the circular perimeter.^10^ Torsion of the left ventricle around its long axis is induced by the contracting fibres of the LV wall.^26^ Because of its direct relation with the orientation of the myofibers, it gives valuable information about the subclinical abnormalities in heart function.^27^ LV torsion is essential for proper myocardial function and it is directly related to the circumferential-longitudinal shear angle.^27^ In the Multi-Ethnic Study of Atherosclerosis including 500 participants with tagged CMR and carotid ultrasound greater IMT was linked with impaired midwall systolic circumferential strain.^25^ Our results are consistent with these findings and beyond midwall strain, participants with the greatest IMT values had significantly lower systolic circumferential strain at the basal and apical levels of the heart even in the fully adjusted model. Our study aimed to examine the atrial alterations, as well. Higher carotid IMT was associated with significantly higher maximal LA volume and lower LA and RA EFs. These changes in atrial structure and function reflect LV dysfunction.

Several mechanisms might account for these associations. One explanation can be that subclinical atherosclerosis might cause damage to the myocardial tissue. Carotid IMT has been shown to be positively associated with coronary artery calcium score and incident coronary heart disease. Coronary atherosclerosis can lead to reduced blood flow to the heart muscles, which can result in abnormal CMR parameters. Atherosclerosis not only affects the large coronary arteries but also impacts the microvasculature within the heart muscle. Chronic exposure to atherosclerosis-related risk factors can also lead to cardiac structural and functional alterations. Prior studies have reported an association between higher carotid IMT, impaired LA function, and epicardial fat accumulation.^28,29^ Moreover, chronic inflammation in patients with higher carotid IMT might cause changes in cardiac mechanics.

The findings of this comprehensive study may suggest the importance of early detection of cardiac remodelling in a reversible phase before the reduction of ventricular EFs. Our results suggest that patients with higher carotid IMT may guide early therapeutic intervention and close follow-up in order to prevent HF occurrence. Further study in this area may help to guide future preventative strategies.

This study has several limitations. First, the effect of asymptomatic coronary artery disease on our findings cannot be excluded. Second, even though we adjusted for potential clinical and laboratory confounders and mediators in the multivariable analyses, unmeasured factors might play a role in the observed associations. Finally, due to its cross-sectional fashion, the cause-effect relationship between the examined associations cannot be confirmed.

## Conclusion

This study reports a comprehensive assessment of the association between carotid IMT and cardiac structure and function in all four chambers as depicted by CMR in the middle-aged population without known overt CV disease. Higher carotid IMT values were linked with LV, RV, LA, and RA enlargement, greater LVM, reduced atrial function, and subclinical LV dysfunction. Since these atherosclerosis-related changes in cardiac structure and function appear much earlier than previously anticipated, our results may facilitate improved preventive actions for the prognostic assessment of higher IMT even in asymptomatic patients.

## Supplementary Material

qyad010_Supplementary_Data

## Data Availability

Publicly available data from the UK Biobank Study were analysed in this study. The datasets are available to researchers through an open application via https://www.ukbiobank.ac.uk/register-apply/.

## Lead Author Biography

Dr. Judit Simon is a radiology resident at Medical Imaging Centre, Semmelweis University, Budapest, Hungary. She completed her M.D. and Ph.D. at Semmelweis University, Budapest, Hungary. Her current research interests include cardiac imaging (coronary computed tomography angiography and cardiac magnetic resonance imaging) as well as interventional radiology.
