# Supplementary material for: Association between subclinical atherosclerosis and cardiac structure and function—results from the UK Biobank Study
Source: Eur Heart J Imaging Methods Pract. 2023 Sep 19;1(2):qyad010. doi: 10.1093/ehjimp/qyad010 (PMC10563379; doi:10.1093/ehjimp/qyad010)
Supplement: qyad010_Supplementary_Data [file qyad010_Supplementary_Data.pdf]

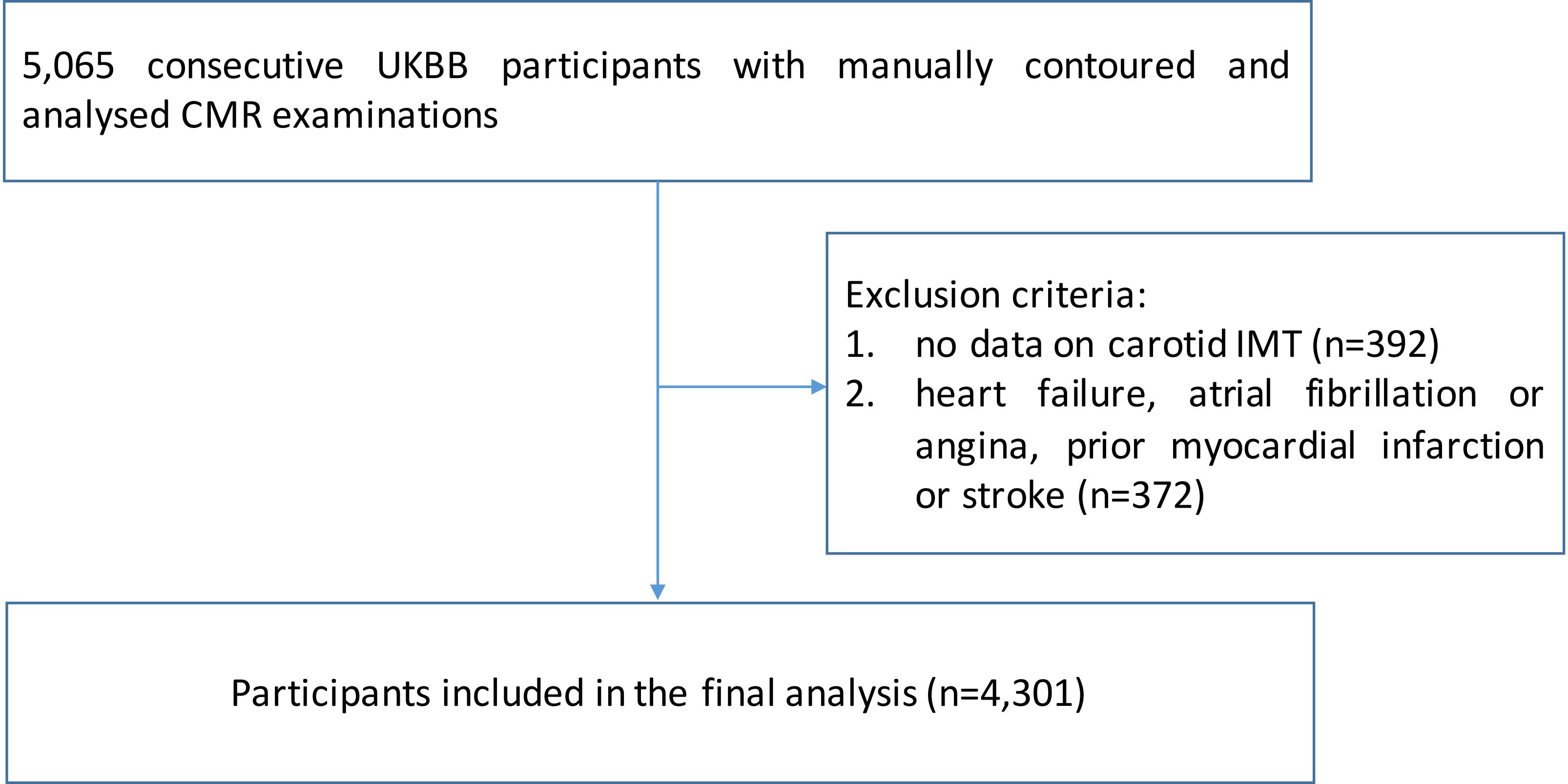
5,065 consecutive UKBB participants with manually contoured and analysed CMR examinations
Exclusion criteria:
1. no data on carotid IMT (n=392)
2. heart failure, atrial fibrillation or angina, prior myocardial infarction or stroke (n=372)
Participants included in the final analysis (n=4,301)
